# Laminin is uniformly expressed in basement membranes surrounding longitudinal axon tracts in the ventral and dorsal nerve cords in *C. elegans*

**DOI:** 10.17912/micropub.biology.001766

**Published:** 2025-08-12

**Authors:** Harald Hutter

**Affiliations:** 1 Biological Sciences, Simon Fraser University, Burnaby, British Columbia, Canada

## Abstract

Laminin is a core basement membrane (BM) component. Mutations in laminin subunits cause a variety of defects including axon navigation defects. We examined the localization of the laminin subunits LAM-3 (laminin αA), EPI-1 (laminin αB) and LAM-2 (laminin γ) in basement membranes surrounding the ventral nerve cord (VNC) and the dorsal nerve cord (DNC) in *C. elegans* . We found that EPI-1 and LAM-2 are uniformly distributed, which suggests that laminins might provide an adhesive substrate for axons but not a directional cue for the positioning of VNC and DNC axon tracts.

**Figure 1. Laminin localization with respect to ventral and dorsal nerve cords:**
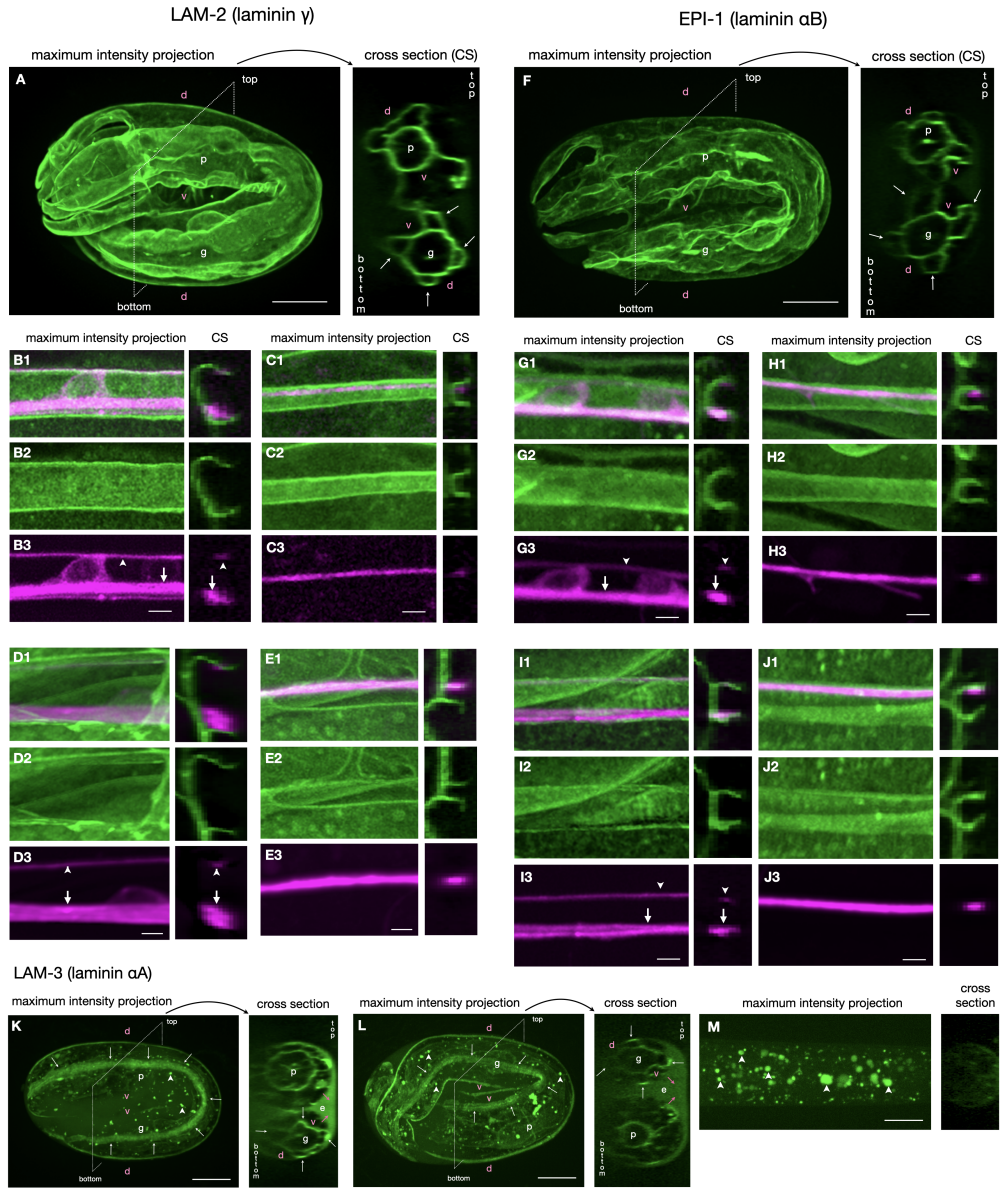
For all panels, images are maximum intensity projections. Anterior is to the left. Scale bar is 10μm for panels A, F, K, L, M and 2μm for all other panels. 'd': dorsal, 'v': ventral, 'p': pharynx, 'g': gut, 'e': extracellular space between embryo and eggshell. Panels: (1) overlay; (2) mNG; (3) *rgef-1 ::DsRed2.* The arrows point to the right axon tract, the arrowheads to the left axon tract. (A-E) LAM-2 ::mNG expression in ventral and dorsal nerve cords. (A) 2-fold embryo, side view. The panneuronal marker is not expressed at this stage. Arrows in the cross-section point to the muscle quadrants. (B) L2 larval VNC. (C) L2 larval DNC. (D) adult VNC. (E) adult DNC. (F-J) EPI-1 ::mNG expression in dorsal and ventral nerve cords. (F) 2-fold embryo, side view. The panneuronal marker is not expressed at this stage. Arrows in the cross-section point to the muscle quadrants. (G) L2 larval VNC. (H) L2 larval DNC. (I) adult VNC. (J) adult DNC *.* (K-M) LAM-3 ::mNG expression. White arrows point to the muscle quadrants. Magenta arrows point to LAM-3 :mNG in the extracellular space (e). Arrowheads point to autofluorescent gut granula. (K) 2-fold embryo, side view. (L) 3-fold embryo, side view. (M) L2 larva, no expression.

## Description

Laminins are heterotrimeric glycoproteins consisting of one α, β and γ subunit (Timpl and Brown, 1994). They form a network within basement membranes (BMs) and are essential for the structural integrity of BMs (Paulsson, 1992; Timpl and Brown, 1996). *C. elegans* has four laminin subunits, LAM-3 /αA, EPI-1 /αB, LAM-1 /β, LAM-2 /γ and can form two distinct laminin isoforms that differ by their α subunit (Huang et al., 2003). Loss of laminin function results in disorganisation of embryonic tissues and cell detachments leading to embryonic lethality (Kao et al., 2006). Partial loss-of-function alleles of *lam-1* or *lam-2* lead to egg-laying defects, uncoordinated movement, and a dumpy body shape due to severe defects in the integrity of the basement membranes (Kao et al., 2006). Mutations in the α and β subunits disrupt cell polarity, cell migrations and axon navigation (Kao et al., 2006). *epi-1* alleles were isolated in genetic screens for mutations causing cell or axon migration defects (Forrester et al., 1998; Forrester and Garriga, 1997).

Laminin expression has been analysed with subunit-specific antibodies and GFP-tagging (Huang et al., 2003; Kao et al., 2006). Laminins are found in all basement membranes surrounding the major tissues in *C. elegans* and expression appears to be uniform (Huang et al., 2003). As expected, the expression patterns of *lam-1* and *lam-2* are identical. Expression starts at the gastrulation stage. The two α subunits show different distribution (Huang et al., 2003). LAM-3 /αA is found in BMs surrounding body wall and pharyngeal muscle as well as the nerve ring and the ventral nerve cord (VNC). EPI-1 /αB is found in all major basement membranes but has not been reported in BMs surrounding axon tracts (Huang et al., 2003). More recently, a large-scale study (Keeley et al., 2020) used CRISPR/Cas9 to tag major BM components and their receptors with mNeonGreen (mNG). In this study LAM-1 , LAM-2 and EPI-1 were found in BMs of muscle, epidermis, pharynx, intestine and nerve ring. LAM-3 was found in all these BMs except for the nerve ring, which is not consistent with the earlier observations using antibodies (Huang et al., 2003). LAM-3 was found to be associated with misguided axons in the VNC (Huang et al., 2003), which could mean that laminin acts an an instructive cue for axon navigation.

We have found that *epi-1 ( rh200 )* mutants have partially penetrant defects in establishing the asymmetry of the VNC axon tract (Taylor and Hutter, 2019) - the right tract has ~50 axons, whereas the left tract has only 4 axons, suggesting a possible function in directional axon outgrowth. We therefore wanted to examine the precise localization of laminin subunits with respect to the position of axons in the VNC as well as the DNC, where axons are asymmetrically localized on the left side of the dorsal midline. We obtained mNG tagged strains for EPI-1 /αB, LAM-3 /αA and LAM-2 /γ and introduced a red panneuronal marker to visualize axons. The LAM-1 ::mNG strain is homozygous lethal, which suggests that the mNG tag interferes with protein function. We therefore did not analyse LAM-1 /β distribution, which is expected to be identical to LAM-2 /γ, since both subunits are present in all laminin molecules. We found that at the 2-fold stage, when axons grow out in the embryo, both EPI-1 /αB and LAM-2 /γ are fairly uniformly expressed in the basement membranes surrounding the VNC and DNC ( Figure 1A and 1F). A somewhat uneven distribution visible in individual cross-sections does not extend for more than a few sections. The same distribution was found in early larval stages and adults ( Figure 1B - 1E, 1G - 1J), suggesting that the initial uniform distribution is maintained through all life stages as the animal grows. This observation suggests that laminin does not act as instructive cue to guide axons and that laminin associated with misguided axons probably did not cause the guidance defect. In 2-fold and 3-fold stage embryos, LAM-3 ::mNG is enriched at the muscle-epidermis attachment interface ( Figure 1K, L) like UNC-52 /Perlecan (Mullen et al., 1999). This aspect of the expression is consistent with the antibody observations (Huang et al., 2003). We observed weak and uniform expression in BMs surrounding the VNC and DNC but no expression at the nerve ring. This is consistent with the LAM-3 ::mNG description from Keeley et al. but not consistent with the antibody staining (Huang et al., 2003). We also observed a strong and uniform mNG signal in the entire extracellular space between embryo and eggshell ( Figure 1K ). This was unexpected, since laminin has no known role outside of the embryo and LAM-2 , which is required to form a functional laminin polymer with LAM-3 , shows no obvious expression in the extracellular space outside the embryo. Expression levels of LAM-3 are substantially lower compared to EPI-1 and LAM-2 . Postembryonically, we cannot detect any LAM-3 ::mNG signal.

The observations with the mNG-tagged strains are mostly consistent with the earlier antibody stainings. A major advantage of antibodies as protein visualization tool is that they can detect the native unmodified protein. The mNG tag could interfere with protein localization and/or function. A major advantage of the mNG tag is the ability to image live animals with intact tissues at high resolution. Antibody stainings require tissue dehydration and fixation, which reduces the resolution and makes it difficult to detect soluble pools of the protein of interest. This might explain, why the extracellular pool of LAM-3 was not detected earlier. Alternatively, the mNG tag might interfere with polarized secretion of LAM-3 towards the interior of the embryo.

Taken together, our data confirm the idea that laminins might have a permissive role in axon navigation in *C. elegans* , e.g. by providing an adhesive substrate that allows axons to attach to the BM. Based on their uniform distribution in BMs surrounding the VNC and DNC they are unlikely to provide directional cues for axon navigation and do not seem to be responsible for the positioning of VNC and DNC axon tracts.

## Methods

**Microscopy**

Animals from a growing population (20˚ C) were immobilized with 10 mM sodium azide and mounted on 2% agarose pads. Animals were imaged on a Zeiss Axioplan II microscope equipped with a Quorum WaveFX spinning disc system (Quorum Technologies, Canada) using a 63x, 1.4 NA objective. Stacks of confocal images with 0.1 to 0.2 μm distance between focal planes were recorded. Resolution in x and y (i.e. pixel size in maximum intensity projections) is 0.1 μm. Volocity software (Quorum Technologies, Canada) was used for image acquisition and processing including iterative deconvolution. Images in the figure are maximum intensity projections and cross-sections.

## Reagents

**Table:** 

Strain	Genotype	Source
VH3020	*lam-2 ( qy20* [ *lam-2 ::mNG+LoxP* ] *)* X *; hdIs36* [ *rgef-1 ::DsRed2* ] IV.	(Keeley et el., 2020) and our lab
VH3022	*lam-3 ( qy28* [ *lam-3 ::mNG+loxP* ] *)* I *; hdIs36* [ *rgef-1 ::DsRed2* ] IV.	(Keeley et el., 2020) and our lab
VH3031	*epi-1 ( qy31* [ *epi-1 ::mNG+loxP* ] *)* IV *; hdIs35* [ *rgef-1 ::DsRed2]. *	(Keeley et el., 2020) and our lab
